# Associations Between Product Type and Intensity of Tobacco and Cannabis Co-use on the Same Day Among Young Adult Smokers: Smartphone-Based Daily-Diary Study

**DOI:** 10.2196/40736

**Published:** 2023-02-20

**Authors:** Nhung Nguyen, Johannes Thrul, Torsten B Neilands, Pamela M Ling

**Affiliations:** 1 Center for Tobacco Control Research and Education University of California San Francisco San Francisco, CA United States; 2 Division of General Internal Medicine University of California San Francisco San Francisco, CA United States; 3 Department of Mental Health Bloomberg School of Public Health Johns Hopkins University Baltimore, MD United States; 4 Sidney Kimmel Comprehensive Cancer Center at Johns Hopkins Baltimore, MD United States; 5 Centre for Alcohol Policy Research La Trobe University Melbourne Australia; 6 Center for AIDS Prevention Studies Division of Prevention Science University of California San Francisco San Francisco, CA United States

**Keywords:** tobacco, cannabis, substance co-use, young adults, intensive longitudinal data, EMA, mHealth, smartphone-based data collection, data collection, smartphone data, substance use

## Abstract

**Background:**

Co-use of tobacco and cannabis is highly prevalent among young US adults. Same-day co-use of tobacco and cannabis (ie, use of both substances on the same day) may increase the extent of use and negative health consequences among young adults. However, much remains unknown about same-day co-use of tobacco and cannabis, in part due to challenges in measuring this complex behavior. Nuanced understanding of tobacco and cannabis co-use in terms of specific products and intensity (ie, quantity of tobacco and cannabis use within a day) is critical to inform prevention and intervention efforts.

**Objective:**

We used a daily-diary data collection method via smartphone to capture occurrence of tobacco and cannabis co-use within a day. We examined (1) whether the same route of administration would facilitate co-use of 2 substances on the same day and (2) whether participants would use more tobacco on a day when they use more cannabis.

**Methods:**

This smartphone-based study collected 2891 daily assessments from 147 cigarette smokers (aged 18-26 years, n=76, 51.7% female) during 30 consecutive days. Daily assessments measured type (ie, cigarette, cigarillo, or e-cigarette) and intensity (ie, number of cigarettes or cigarillos smoked or number of times vaping e-cigarettes per day) of tobacco use and type (ie, combustible, vaporized, or edible) and intensity (ie, number of times used per day) of cannabis use. We estimated multilevel models to examine day-level associations between types of cannabis use and each type of tobacco use, as well as day-level associations between intensities of using cannabis and tobacco. All models controlled for demographic covariates, day-level alcohol use, and time effects (ie, study day and weekend vs weekday).

**Results:**

Same-day co-use was reported in 989 of the total 2891 daily assessments (34.2%). Co-use of cigarettes and combustible cannabis (885 of the 2891 daily assessments; 30.6%) was most commonly reported. Participants had higher odds of using cigarettes (adjusted odds ratio [AOR] 1.92, 95% CI 1.31-2.81) and cigarillos (AOR 244.29, 95% CI 35.51-1680.62) on days when they used combustible cannabis. Notably, participants had higher odds of using e-cigarettes on days when they used vaporized cannabis (AOR 23.21, 95% CI 8.66-62.24). Participants reported a greater intensity of using cigarettes (AOR 1.35, 95% CI 1.23-1.48), cigarillos (AOR 2.04, 95% CI 1.70-2.46), and e-cigarettes (AOR 1.48, 95% CI 1.16-1.88) on days when they used more cannabis.

**Conclusions:**

Types and intensities of tobacco and cannabis use within a day among young adult smokers were positively correlated, including co-use of vaporized products. Prevention and intervention efforts should address co-use and pay attention to all forms of use and timeframes of co-use (eg, within a day or at the same time), including co-use of e-cigarettes and vaporized cannabis, to reduce negative health outcomes.

## Introduction

Co-use of tobacco and cannabis is highly prevalent among young US adults. National data indicates that 21% of the general population of young adults has used both tobacco and cannabis in the past 30 days [1]. The use of cannabis is associated with persistent cigarette smoking and may pose a barrier to successful tobacco cessation [2,3]. At the person level, combined use of tobacco and cannabis can increase the risk for addiction and negative health outcomes (eg, mental health and respiratory problems) among people who co-use both products compared to those who use only a single substance [4,5]. This public health impact from co-use underscores the need to prevent this behavior during young adulthood. However, much remains unknown about co-use of tobacco and cannabis at both the personal level (ie, comparing people who co-use to those using a single substance) and at the event level (eg, comparing co-use to single-substance use within a day), in part due to challenges in measuring this complex behavior [6,7].

At the event level, the inherent complexity of co-use behavior includes a variety of products and timeframes, which adds extra burden to assessment and intervention [6]. People can use both substances in any combination of forms across the wide array of tobacco and cannabis products available on the marketplace [8]. While co-use is commonly defined in survey research as the use of both tobacco and cannabis within a month or year, it can also occur in a shorter timeframe (eg, within the same occasion or day). Studies indicate that the extent to which individuals use tobacco and cannabis closely in time is associated with more cigarettes smoked per day, greater nicotine dependence, and worse physical and mental functioning [9-11]. In addition, exposure to toxicants may vary by route of coadministration (eg, smoking vs vaping), posing differential health impacts [5]. Smoking both tobacco and cannabis is a well-known route, including the use of “blunts” (cannabis rolled in a cigar leaf for smoking), “spliffs” (combining cannabis and loose-leaf tobacco in a joint), or “chasing” (smoking cigarettes after smoking cannabis). A newer route of co-use is with vaporized products, in which liquid- or leaf-vaporizing devices are used to deliver both nicotine and tetrahydrocannabinol (THC—the main psychoactive component in cannabis), sometimes with the same device, on the same occasion, or in quick succession [12]. As such, understanding co-use among young adults at the event level, taking into account specific products and timeframes, is critical to inform prevention and intervention efforts [7].

Existing evidence at the event level, however, has predominantly focused on co-use in general (eg, any tobacco and cannabis) or has been limited to only combustible products (eg, blunts). In addition, co-use is mostly measured as any use of both tobacco and cannabis in the past 30 days, and little is known about intensity of co-use (defined in this study as quantity of tobacco and cannabis use within a day). Studies are lacking that address co-use via newer products and in shorter timeframes, yet these patterns of use may result in greater substance use and associated health impacts. Cross-sectional surveys and retrospective behavioral measures used in prior research have asked few questions about the nuances of co-use [6,7]. Newer data collection methods (eg, daily-diary assessments and ecological momentary assessments) have the potential to capture occurrence of tobacco and cannabis co-use within a day or moment and generate a richer picture of the behavior [7]. Using this approach, a few studies have indicated that cannabis use increased the odds of cigarette use on the same day [13] or within 4-hour windows [14,15] and that same-day co-use was more prevalent among young sexual-minority adults than their heterosexual peers [10]. These studies, however, have not examined the intensity of co-use within a day as well as co-use of noncombustible products (eg, e-cigarettes and vaporized cannabis). A better understanding of co-use at the event level, including whether and how types and intensity of cannabis use would drive tobacco use within a day, may be beneficial in developing interventions targeting young adults with problematic use of both substances.

To address the aforementioned gaps in knowledge of co-use of tobacco and cannabis at the day level, we analyzed smartphone-based daily assessment data collected among 147 young adult cigarette smokers during 2016 and 2017. We examined day-level associations between types (ie, combustible, vaporized, and edible) and intensity (ie, number of times) of cannabis use and types of tobacco product use (ie, cigarettes, cigarillos, and e-cigarettes). Based on the aforementioned research [10,13-15], we hypothesized that (1) participants would use more tobacco on a day when they use more cannabis; and (2) the same route of administration would facilitate co-use of 2 substances (eg, participants would smoke/vape tobacco on a day when they smoke/vape cannabis, respectively).

## Methods

### Study Design

This study analyzed daily assessments from a smartphone-based study conducted in California during 2016 and 2017. The study procedure was described in detail elsewhere [10]. Initially, participants completed a baseline survey on their demographics and substance use history. They were then trained on use of the study app to collect data every day for a 30-day period. Each day, participants were prompted between 10 and 11 AM to complete a daily assessment reporting their use of tobacco and cannabis on the entire previous day, including substance use occurrences late at night. To increase participant study compliance and retention, incentives were contingent on level of data-collection completion.

### Ethics Approval

Electronic informed consent was obtained from all participants. The study was approved by the University of California San Francisco Institutional Review Board (15-18033).

### Study Participants

Participants were recruited through social media and online advertisements (eg, Facebook and Craigslist). To conduct a nested qualitative substudy, participants were also recruited via the websites of sexual-minority youth organizations, and we oversampled women identifying as a sexual minority. Eligible participants were aged 18 to 26 years, had smoked at least 100 cigarettes in their lifetime, and currently smoked at least one cigarette per day at least 3 days per week. Since the parent study focused on cigarette smoking, cannabis use was not part of the inclusion criteria. Of 184 participants who completed the baseline assessments, 147 who completed at least one daily assessment were included in the analytic sample. There was no statistical difference between the analytic sample (n=147) and those who were excluded from the analysis (n=37) in baseline characteristics (ie, age, sex, educational attainment, race, ethnicity, and past-30-day use of tobacco and cannabis). Baseline characteristics of the study sample are presented in Table 1. The sample had a mean age of 22.7 (SD 2.4) years, 51.7% (76/147) of participants were female, 40.8% (60/147) of participants were non-Hispanic White, and 76.9% (113/147) of participants were currently in college or had a college degree or higher. At baseline, a majority of participants reported past-30-day use of cannabis (96/147, 65.3%) and alcohol (136/147, 92.5%). We included 51 participants who did not report past 30-day use of cannabis at baseline in our sample, since these participants could report co-use during the daily-diary period (and n=7 did), allowing for comparison between co-use and single-substance use within a day.

**Table 1:** Sample characteristics.

Characteristics			Total (n=147)
	Age (years), mean (SD)		22.7 (2.4)
**Sex at birth, n (%)**			
	Male		71 (48.3)
	Female		76 (51.7)
**Race, n (%)**			
	Non-Hispanic White		60 (40.8)
	Non-Hispanic Asian		30 (20.4)
	Hispanic		31 (21.1)
	Other/multiracial		17 (11.6)
**Education, n (%)**			
	Less than college		33 (22.5)
	College or higher		113 (76.9)
**Past 30-day substance use at baseline, n (%)**			
	**Tobacco use**		
		Cigarettes	145 (98.6)
		e-Cigarettes	47 (32)
	Cannabis use		96 (65.3)
	Alcohol use		136 (92.5)

### Measures

#### Outcome Variables (Type and Intensity of Tobacco Use)

For each day, participants reported whether they used cigarettes, cigarillos, or e-cigarettes. These binary variables (yes/no) indicated types of tobacco used in each daily assessment. We examined cigarillos rather than other types of cigars since cigarillos are the most common cigar type used by young adults [16]. Regarding intensity of tobacco use, participants were asked, “Yesterday: How many [cigarettes, cigarillos] used?” and “How many times [disposable e-cigarettes, rechargeable e-cigarettes, tanks, or pod-mods] used?” We assessed tobacco products and different types of e-cigarettes separately. These types of devices included 4 generations of e-cigarettes available on the marketplace at the time of the study (eg, the first generation refers to disposable e-cigarettes, the second generation refers to rechargeable e-cigarettes, the third generation refers to tank devices, and the fourth generation refers to pod-mods). A total intensity of e-cigarette use for each day was calculated by summing the intensities of using all 4 types of e-cigarettes. To not overburden participants, response options provided categories of increasing intensity of use of each product (ie, 0, 1, 2-5, 6-10, 11-15, 16-20, 21-30, and ≥31 cigarettes, cigarillos, or times vaping e-cigarettes per day).

### Independent Variables (Type and Intensity of Cannabis Use)

For each day, participants were asked, “How many times did you use marijuana or hash?” Answer options ranged continuously from 0 to 7 or more times. Those who reported any cannabis use were then asked, “How did you use marijuana or hash?” with answer options including smoking, vaping, and edibles. While we asked about intensity of use of cannabis in general, we did not ask about intensity of use of each cannabis product separately, to avoid overburdening participants. As such, depending on a participant’s interpretation, a smoking occasion of combustible cannabis, a hit of a cannabis vaporizer, or consuming 1 edible may have been considered as a single occasion of cannabis use in our study.

#### Covariates

Demographic characteristics were collected at baseline. Age was calculated based on self-reported date of birth. Sex assigned at birth was measured as female or male. Race/ethnicity was categorized into 4 groups: non-Hispanic White, non-Hispanic Asian, Hispanic, and other/multiracial. Educational attainment was dichotomized as “less than college” and “college or higher,” since having a college education is associated with tobacco and cannabis use among young adults [17]. Participants also reported alcohol use (yes/no) in each daily assessment. A dummy variable was created to indicate the study day of each daily assessment, ranging from day 1 to day 30. As use of substances may be different between weekends and weekdays [18], another dummy variable was created to indicate weekend or weekday.

### Statistical Analyses

Statistical analyses were performed using Stata (version 15; Stata Corp). Descriptive statistics of sample characteristics at baseline and substance use in daily assessments were summarized. First, to examine associations of type of tobacco and cannabis co-use on the same day, we fitted multilevel logistic regression models examining associations of use of cannabis products (combustible, vaporized, and edible) with each of the binary outcomes (ie, any use of cigarettes, cigarillos, or e-cigarettes on a given day). Second, to examine associations between intensities of tobacco and cannabis use on the same day, we fitted multilevel mixed-effects ordered logistic regression models examining intensity of cannabis use (ie, number of times using cannabis on a given day) with each of the ordinal outcomes (ie, numbers of cigarettes or cigarillos smoked and number of times using e-cigarettes on a given day) [19]. The models also included random intercepts for participants to control for variation in tobacco use intensity attributable to individual participants.

The variable of intensity of cannabis use was decomposed into 2 elements: personal mean (ie, average intensity of cannabis use for each participant, indicating comparisons between participants, in other words, between-person effects), and deviation (ie, the difference between intensity in a particular daily observation and the personal mean, indicating comparisons across study days within a certain participant, that is, within-person effects) [20]. For each ordinal outcome, the proportional odds assumption was checked by fitting a generalized multinomial logit model and comparing its likelihood ratio to that of the ordinal model [19]; this assumption was satisfied for all the models. All models controlled for demographic covariates, day-level alcohol use [21], and time effects (ie, study day and weekend vs weekday). All tests were 2-tailed with a significance level of α<.05. The analyses were not preregistered and thus the results should be considered exploratory.

## Results

### Daily Assessments of Tobacco and Cannabis Use

During the 30-day study period, 147 participants completed an average of 19.7 (SD 9.6) daily assessments with a completion rate of 65.6% (2891 completed assessments of 4410 prompted assessments). Table 2 describes reports of tobacco and cannabis use among the total of 2891 daily assessments. Co-use was reported in 989 daily assessments (34.2%), while use of tobacco without cannabis was reported in 1501 daily assessments (51.9%). The most common intensity of cigarette use reported in the daily assessments was smoking 2 to 5 cigarettes per day. Not using at all was reported the most in the daily assessments for cigarillos, e-cigarettes, and cannabis. On the days when participants used these products, the common intensities of use were smoking 2 to 5 cigarillos per day, vaping e-cigarettes 2 to 5 times per day and using cannabis once a day.

Table 3 presents same-day co-use in terms of combinations of specific products. The most commonly used tobacco product was cigarettes (2407 of 2891 assessments, 83.3%), while combustible cannabis was the most common type of cannabis use (1040 of 2891 assessments, 36%). The 3 most common product combinations on the same day were cigarettes and combustible cannabis (885 of 2891 assessments, 30.6%), cigarillos and combustible cannabis (197 of 2891 assessments, 6.8%), and cigarettes and vaporized cannabis (147 of 2891 assessments, 5.1%).

**Table 2:** Daily assessments of substance use among young adult smokers.

Substance use assessments		Assessments (n=2891), n (%)
**Daily assessments**		
	Use of tobacco only	1501 (51.9)
	Use of cannabis only	145 (5)
	Use of both substances	989 (34.2)
	No use	251 (8.7)
	Missing data	5 (0.2)
**Number of cigarettes smoked in a day**		
	0	483 (16.7)
	1	303 (10.5)
	2-5	1250 (43.2)
	6-10	644 (22.3)
	11-15	145 (5)
	16-20	61 (2.1)
	21-30	2 (0.1)
	≥31	3 (0.1)
**Number of cigarillos smoked in a day**		
	0	2682 (92.8)
	1	85 (2.9)
	2-5	110 (3.8)
	6-10	11 (0.4)
	11-15	3 (0.1)
**Number of times vaping e-cigarettes in a day**		
	0	2664 (92.2)
	1	17 (0.6)
	2-5	113 (3.9)
	6-10	52 (1.8)
	11-15	22 (0.8)
	16-20	15 (0.5)
	21-30	7 (0.2)
	≥31	1 (<0.1)
**Number of times using cannabis in a day**		
	0	1754 (60.7)
	1	351 (12.1)
	2	255 (8.8)
	3	231 (8)
	4	131 (4.5)
	5	77 (2.7)
	6	17 (0.6)
	≥7	75 (2.6)
Daily assessments with alcohol use		1032 (35.7)
Daily assessments on weekend		804 (27.8)
Daily assessments on weekday		2087 (72.2)

**Table 3:** Same-day co-use of specific tobacco and cannabis products among young adult smokers (n=2891 assessments). Proportions were calculated as frequency of a given product combination out of the total daily assessments.

Types	Any cannabis (n=1136, 39.3%), n (%)	Combustible cannabis (n=1040, 36%), n (%)	Vaporized cannabis (n=190, 6.6%), n (%)	Edible cannabis (n=36, 1.3%), n (%)
Any tobacco (n=2490, 86.1%)	989 (34.2)	915 (31.7)	151 (5.2)	28 (1)
Cigarette (n=2407, 83.3%)	956 (33.1)	885 (30.6)	147 (5.1)	27 (0.9)
e-Cigarette (n=240, 8.3%)	101 (3.5)	79 (2.7)	39 (1.4)	4 (0.1)
Cigarillo (n=209, 7.2%)	197 (6.8)	197 (6.8)	8 (0.3)	5 (0.2)

### Associations Between Type of Cannabis and Tobacco Products Used on the Same Day

Results from the mixed-effects models are shown in Table 4. Participants had higher odds of reporting using cigarettes (adjusted odds ratio [AOR] 1.92, 95% CI 1.31-2.81) and cigarillos (AOR 244.29, 95% CI 35.51-1680.62) on days when they used combustible cannabis. Notably, participants had higher odds of using e-cigarettes on days when they used vaporized cannabis (AOR 23.21, 95% CI 8.66-62.24). It should be noted that the CIs for cigarillos and e-cigarettes were quite wide due to the small number of daily assessments with use of these products. In addition, participants had higher odds of smoking cigarettes on days with alcohol use (AOR 2.73, 95% CI 1.99-3.76). The study day was negatively associated with the odds of smoking cigarettes (AOR 0.96, 95% CI 0.95-0.98) and cigarillos (AOR 0.96, 95% CI 0.92-0.99). Older participants (vs younger peers) had higher odds of reporting cigarette smoking (AOR 1.31, 95% CI 1.10-1.57), while Hispanic participants (vs non-Hispanic White peers) had higher odds of reporting cigarillo smoking (AOR 22.93, 95% CI 3.30-159.49); however, this estimate was very wide due to a small number of cigarillo-use reports.

**Table 4:** Day-level associations between tobacco use product types (outcomes) and cannabis use product types (independent variables) among young adult cigarette smokers (n=2891 assessments), controlled for time-varying covariates (day-level) and demographic covariates (participant-level). The outcomes were binary variables (ie, any use of a tobacco product on a given day). All variables were included in a mixed-effects logistic regression model for each outcome.

Independent variables				Model 1: cigarette smoking AOR^a^ (95% CI)		*P* value			Model 2: cigarillo smoking AOR (95% CI)				*P* value				Model 3: e-cigarette vaping AOR (95% CI)				*P* value		
**Type of cannabis use**																							
	Combustible cannabis			1.92 (1.31-2.81)		.001			244.29 (35.51-1680.62)				<.001				1.84 (0.93-3.67)				.08		
	Vaporized cannabis			1.58 (0.83-3.03)		.17			0.83 (0.30-2.25)				.71				23.21 (8.66-62.24)				<.001		
	Edible cannabis			0.58 (0.21-1.63)		.30			1.18 (0.28-5.02)				.82				4.90 (0.90-26.58)				.07		
**Time-varying covariates**																							
	Alcohol use			2.73 (1.99-3.76)		<.001			0.91 (0.51-1.63)				.76				1.63 (0.90-2.93)				.10		
	Weekend vs weekday			0.85 (0.65-1.12)		.26			1.02 (0.60-1.72)				.96				0.79 (0.48-1.31)				.36		
	Study day			0.96 (0.95-0.98)		<.001			0.96 (0.92-0.99)				.01				1.00 (0.97-1.03)				.84		
**Demographic covariates**																							
	Age			1.31 (1.10-1.57)		.003			1.00 (0.75-1.33)				.98				0.92 (0.69-1.22)				.55		
	Female vs male			1.06 (0.48-2.34)		.88			2.32 (0.57-9.44)				.24				0.84 (0.24-2.98)				.78		
	Education (college or higher vs less)			0.57 (0.20-1.62)		.29			0.70 (0.11-4.52)				.71				0.49 (0.10-2.52)				.40		
	**Race (reference non-Hispanic White)**																						
		Non-Hispanic Asian	1.47 (0.51-4.20)		.48			6.84 (0.90-52.23)				.06				2.01 (0.38-10.64)				.41			
		Hispanic	2.85 (0.88-9.25)		.08			22.93 (3.30-159.49)				.002				0.82 (0.14-4.94)				.83			
		Other/multiracial	1.19 (0.41-3.43)		.75			4.29 (0.65-28.21)				.13				0.77 (0.13-4.56)				.78			

^a^AOR: adjusted odds ratio.

### Associations Between Intensity of Cannabis and Tobacco Use on the Same Day

Results from the multilevel ordinal models are shown in Table 5. Participants had higher odds of reporting a greater intensity of using cigarettes (AOR 1.35, 95% CI 1.23-1.48), cigarillos (AOR 2.04, 95% CI 1.70-2.46), and e-cigarettes (AOR 1.48, 95% CI 1.16-1.88) on days when they used more cannabis. In addition, alcohol use on a given day was positively associated with intensity of cigarette use (AOR 1.41, 95% CI 1.35-1.49). Participants with higher average intensity of cannabis use had higher average intensity of cigarillo use (AOR 3.72, 95% CI 2.41-5.73). The study day was negatively associated with intensity of smoking cigarettes (AOR 0.97, 95% CI 0.96-0.98) and cigarillos (AOR 0.94, 95% CI 0.91-0.97), meaning that intensity of smoking cigarettes and cigarillos decreased slightly over the study period. Those with education attainment of college or higher reported lower intensity of cigarette smoking (AOR 0.30, 95% CI 0.09-0.97), while older participants reported higher intensity of cigarette smoking (AOR 1.52, 95% CI 1.22-1.89). Hispanic participants (vs non-Hispanic White peers) had higher odds of reporting higher intensity of cigarillo smoking (AOR 11.46, 95% CI 1.82-72.36); however, this estimate was very wide due to a small number of cigarillo-use reports.

**Table 5:** Day-level associations between tobacco use intensity for different products (as outcomes) and intensity of cannabis use (as independent variables) among young adult cigarette smokers, controlling for time-varying (day-level) and demographic (participant-level) covariates (n=2891 assessments). The outcomes were categorical variables (ie, 0, 1, 2-5, 6-10, 11-15, 16-20, 21-30, and ≥31 cigarettes, cigarillos, or times vaping e-cigarettes in a given day). All variables were included in a multilevel mixed-effects ordered logistic regression model for each outcome.

Independent variables				Model 1: cigarette smoking intensity AOR^a^ (95% CI)		*P* value		Model 2: cigarillo smoking intensity AOR (95% CI)		*P* value		Model 3: e-cigarette vaping intensity AOR (95% CI)		*P* value
**Intensity of cannabis use**														
	Intensity of cannabis use in a given day			1.35 (1.23-1.48)		<.001		2.04 (1.70-2.46)		<.001		1.48 (1.16-1.88)		.001
	Personal mean of cannabis use intensity			1.05 (0.78-1.42)		.75		3.72 (2.41-5.73)		<.001		1.16 (0.75-1.78)		.51
**Time-varying covariates**														
	Intensity of alcohol use in a given day			1.41 (1.35-1.49)		<.001		1.02 (0.90-1.15)		.77		1.04 (0.93-1.16)		.48
	Personal mean of alcohol use intensity			0.95 (0.63-1.43)		.80		0.86 (0.44-1.66)		.65		1.08 (0.59-1.99)		.80
	Weekend vs weekday			0.95 (0.80-1.12)		.52		1.05 (0.67-1.65)		.84		0.86 (0.57-1.30)		.48
	Study day			0.97 (0.96-0.98)		<.001		0.94 (0.91-0.97)		<.001		1.01 (0.99-1.04)		.25
**Demographic covariates**														
	Age			1.52 (1.22-1.89)		<.001		1.00 (0.72-1.37)		.98		0.82 (0.60-1.14)		.24
	Female vs male			0.45 (0.18-1.13)		.09		3.89 (0.95-15.91)		.06		1.06 (0.28-4.04)		.94
	Education (college or higher vs less)			0.30 (0.09-0.97)		.045		0.82 (0.15-4.57)		.82		0.67 (0.12-3.81)		.66
	**Race (reference: non-Hispanic White)**													
		Non-Hispanic Asian	1.05 (0.30-3.65)		.94		6.39 (0.90-45.55)		.06		1.35 (0.23-7.82)		.74	
		Hispanic	1.58 (0.44-5.61)		.48		11.46 (1.82-72.36)		.01		0.31 (0.04-2.35)		.26	
		Other/multiracial	0.72 (0.19-2.73)		.63		2.96 (0.37-23.54)		.31		0.60 (0.09-4.07)		.60	

^a^AOR: adjusted odds ratio.

## Discussion

### Principal Results

This study is one of very few examining young adult co-use of tobacco and cannabis within shorter timeframes (ie, a day) than the typical survey measure of past-30-day use, and it is among the first to examine same-day co-use of tobacco and cannabis products that are not smoked (ie, e-cigarettes and vaporized cannabis), including day-level intensity of co-use. The main findings were as hypothesized and showed that the more cannabis participants reported using on a given day, the greater the intensity of tobacco product use (cigarettes, cigarillos, and e-cigarettes). Notably, participants reported smoking cigarettes or cigarillos on the days they smoked cannabis, and vaping e-cigarettes on the days they vaped cannabis, indicating the same routes of administration may play a role in facilitating same-day co-use.

### Comparison With Prior Work

Since traditional measures are insufficient to fully capture and monitor co-use of tobacco and cannabis, recent research called for more accurate measures of this behavior [6] and highlighted the potential of digital health applications for collecting fine-grained data and specifying co-use patterns [7]. As a methodological example, our study used a daily-diary design and smartphone-based data collection to generate intensive longitudinal data on co-use patterns on a daily basis over 30 consecutive days, providing a nuanced understanding of the extent of co-use within a day. Another strength of this study was an examination of use of a variety of tobacco and cannabis products, including co-use of vaporized products (ie, e-cigarettes and vaporized cannabis), for which more evidence is needed. In addition to our smartphone-based daily assessment method, future research should also consider using other mobile-data collection methods (eg, ecological momentary assessments and mobile sensors) that may more comprehensively assess co-use of tobacco and cannabis [6,7]. Furthermore, while there are only a handful of studies, including this study, that have directly examined co-use as the focal outcome, many prior studies indirectly addressed co-use by adjusting for use of both tobacco and cannabis in the same analytic models. Systematic review or meta-analysis of both direct and indirect evidence may be warranted to provide comprehensive insights on co-use and its effects.

The positive associations between use of the same types of tobacco and cannabis products on the same day indicate that there may be behavioral cues from shared routes of administration that may facilitate co-use of tobacco and cannabis (eg, smoking or vaping one substance triggers smoking or vaping the other) [22]. Indeed, a combination of cigarettes and combustible cannabis was the most common same-day co-use pattern in our sample of young adult cigarette smokers. It was also the most common pattern of past-30-day and past-year co-use found in other samples of young adults [8,11]. In addition to well-documented co-use patterns via smoking (eg, cigarettes/cigarillos and combustible cannabis), we also found that participants reported vaping e-cigarettes more on the days when they vaped cannabis. This finding, coupled with the high prevalence of vaping among young populations, indicates that more attention to emerging co-use patterns via vaping is needed [12,23]. Previous research has reported vaping-related harms among covapers, such as lung impairments [24] and increased odds of having COVID-19 symptoms and diagnoses [25]. Further investigation of tobacco and cannabis covaping among young adults and its health consequences is warranted. Moreover, our participants also reported using other product combinations across the spectrum of tobacco and cannabis products, underlining the heterogeneity of co-use patterns. Further exploration of unique reasons and contexts for different patterns of co-use would help to identify targets for tailored prevention and treatment strategies.

While one might expect a potential drug substitution effect, in which people use cannabis as a substitute for tobacco [26], our finding of positive associations between intensity of tobacco and cannabis use on the same day suggests the substitution effect did not occur in our sample of young adult smokers. Instead, as explained by the theory of synergistic effects, individuals may use 2 substances at the same time or use 1 substance under the effect of the other to amplify positive effects or counteract negative effects between nicotinic and endocannabinoid systems [22]. Relatedly, shared contexts (eg, being with friends and socializing) may also facilitate intensity of same-day co-use of tobacco and cannabis [27,28]. In addition, our participants with higher average intensity of cannabis use also reported higher average intensity of cigarillo use. This finding could be due to our participants using cigarillos for blunt smoking. Although we did not directly ask about blunt use in the daily assessments, previous studies indicated that young adults perceived cigarillos were frequently used for blunts [29,30]. We also found that participants smoked more cigarettes on the days when they drank alcohol. This finding could be explained by well-known rewarding effects when cigarettes and alcohol are used together [13,31-33].

In addition, several subgroups in our sample demonstrated greater average intensity of tobacco use. Participants who were older and had less than a college education reported greater intensity of cigarette smoking, whereas Hispanic participants reported a greater intensity of cigarillo use. These findings are consistent with previous research documenting high prevalence of tobacco use in these subgroups [17,34,35]. Interestingly, participants’ use of cigarettes and cigarillos decreased over the study period. This may be due to a Hawthorne effect or other impacts of research participation, as the process of reporting on their own behaviors may induce reflection and influence participants’ behaviors [36]. To our knowledge, this reactivity effect was rarely observed in previous research. Future studies using experience-sampling methods (eg, ecological momentary assessments or daily diaries) should explore reactivity effects and potential impacts on behavioral outcomes.

### Study Implications

Collectively, our study has implications for efforts to support smoking cessation among young adults. As co-use of tobacco and cannabis was common, and this may increase harm and addiction, smoking cessation programs may need to address co-use of multiple tobacco products or tobacco and cannabis to improve efficacy with this age group. Most available interventions to reduce tobacco use in young people may not address engaging in co-use [7,37]. A recent study found that when young people reduced their tobacco use, their cannabis use also decreased, suggesting the potential benefits of dual cessation treatment for co-users [38]. In addition, treatment strategies should be expanded to include co-use of nonsmoking products to meet cessation needs of covapers [7,39]. As such, tailored interventions that adapt supports to individuals’ co-use patterns may be more effective for reducing the use of both substances. Moreover, tailored interventions may be needed to reach those with high rates of co-use, such as those without college education or those who identify as Hispanic.

### Limitations

Several limitations ought to be considered. The data were collected during 2016 and 2017. Since then, there have been rapid changes in public policy related to both tobacco and cannabis, in patterns of use (eg, increasing use of vaporization devices), in cannabis legalization, and in product availability in the marketplace. As such, more recent data are needed to replicate our findings. The convenience sampling procedure via online recruitment in California and the oversampling of young sexual minority adults limit our study’s generalizability to other young adult samples or geographic regions. While co-use of tobacco and cannabis is common among smokers, our sample included a minority of young adults who did not report past-30-day cannabis use at baseline; further research should examine co-use of tobacco and cannabis among young adults who report recent use of both substances. Although using categories for intensity of use of tobacco products provided a general measure of increasing intensity, this may result in limitations to interpretation of actual effects for each product, given that the increase of using e-cigarettes from, for example, one time per day to 2 to 5 times per day may be different from the increase of smoking from one to 2 to 5 cigarillos per day. Data on cigar use were not collected, and simultaneous use of tobacco and cannabis and their overlapping effects were not directly assessed in our study. Moreover, data on timing or ordering in use of tobacco and cannabis were not collected; thus, we could not identify temporal relationships in use of these substances. Likewise, we did not collect data on cannabis concentrations and specific intensity of use by type of cannabis. The use of concentrated cannabis could impact the same-day co-use of tobacco and cannabis in meaningful ways depending on complementing versus supplementing behaviors. In addition, our participants were not trained in defining intensity of cannabis use and the meaning of “times of cannabis use” may vary depending on personal definitions of use sessions and types of cannabis use. Future research should consider collecting these data and developing more accurate measures of daily use of tobacco and cannabis in order to provide a better understanding of co-use. Missing data due to participants’ compliance with daily assessments may impact the study’s internal validity; however, our compliance rate is within the range of previous studies using the same data collection methods [10,13-15] and the models in our analysis are generally robust to missing data under the missing-at-random assumption [40,41].

### Conclusion

By using smartphone-based daily assessments, this study identified a substantial correlation of product types and intensities of tobacco and cannabis co-use at the day level, with young adults reporting more tobacco use on days when they used more cannabis, including same-day co-use of e-cigarettes and vaporized cannabis. Future research and interventions should address co-use in all forms, especially co-use via new products and in short timeframes, to better prevent and reduce use of both tobacco and cannabis and related health impacts among young people.

## Abbreviations

AOR: adjusted odds ratio
